# Migration of adult children and mental health of older parents ‘left behind’: An integrative review

**DOI:** 10.1371/journal.pone.0205665

**Published:** 2018-10-22

**Authors:** Deependra Kaji Thapa, Denis Visentin, Rachel Kornhaber, Michelle Cleary

**Affiliations:** School of Health Sciences, College of Health and Medicine, University of Tasmania, Sydney, NSW, Australia; Yokohama City University, JAPAN

## Abstract

**Background:**

Although a number of studies have examined the effect of the out-migration of children on the mental health of ‘left behind’ elderly parents, research on the consequences of children’s migration on the mental health and well-being of elderly parents left behind is inconclusive and a systematic review is warranted.

**Objectives:**

To identify the association between the left behind or empty nest status and the mental health of older parents, and to identify common risk factors for poor mental health among those left behind.

**Methods:**

Online databases CINAHL, PsycINFO, PubMed, Scopus and ProQuest were searched for research (2000-September 2017) that focused on the relationship between the migration of adult children and the mental health of the older parents (≥50 years) left behind. The JBI Checklist for Analytical Cross Sectional Studies was used to assess the methodological quality of the articles.

**Results:**

25 articles met the inclusion criteria. The studies identified that left behind older parents had higher levels of mental health problems compared to non-left behind. Left behind parents had higher depressive symptoms, higher levels of loneliness, lower life satisfaction, lower cognitive ability and poorer psychological health. A number of risk factors were identified for mental health disorders among the left behind parents, which included living arrangements, gender, education, income, physical health status, physical activity, family and social support, age, rural residence and frequency of children’s visit.

**Conclusions:**

This review synthesised the various studies related to the mental health of left behind parents, advancing the theoretical and empirical understanding of the implications of out-migration of adult children on the psychological health and well-being of older parents. More responsive preventive measures and effective management approaches are required for this vulnerable cohort.

## Introduction

Over the past decade, there has been a significant increase in both international and internal migration rates. There is an increasing trend in the flow of rural surplus labour to big cities due to an imbalance in economic development between rural and urban areas, exacerbated by globalization and urbanization. Globally there are an approximately 232 million international migrants and 740 million internal migrants [1]. Potential migrants are more likely to be male, young, single and have completed secondary education [2]. The out-migration of young adults from the household results in children and older family members being ‘left behind’. Studies concerning the effects of migration on health and well-being often focus on migrants themselves with the families left behind receiving limited attention [3]. Studies focusing on the left behind often consider the children [4, 5, 6] and spouse [7, 8] of migrants, ignoring the left behind older family members themselves.

### The ‘left behind’ and ‘empty nest’ parents

Left behind parents are those who are living in the originating country or place of residence with one or more biological or adopted children emigrated. Older adults without living child(ren) are not considered at risk of being ‘left behind’. When a household consists of only older adult(s) after children leave the home, it is called the ‘empty nest’ although some studies also use the term to include childless households. Hence, ‘empty nest older adults’ live alone or only with a spouse and may experience anxiety, depression, guilt, and loneliness; the so-called ‘empty nest syndrome’ [9, 10].

While both terms ‘left behind’ and ‘empty nest’ parents portray similar meanings, there are some important distinctions. Firstly, older adults who do not have a child might not be considered as ‘left behind’, but may fall into the ‘empty nest’ category if living alone or with a spouse. Secondly, when one or more children leave the household the parents are ‘left behind’ irrespective of the living arrangement and household structure. However, elders who live alone or with their spouse only are defined as empty nest elders, while those who live with one or more children are non-empty nest elders, despite the fact that the parent may have some children who have migrated. The focus of this paper is on the impact of out-migration of children on the mental health of the older parents left behind and hence consider studies that use either terms.

### Mental health of left behind parents

A number of studies have explored the influence of adult children’s migration on the health of older parents left behind, with some studies reporting a significant adverse effect on their mental health. Out-migration of young people has negative consequences for ageing parents, with loneliness, isolation and loss of basic support [11]. In Mexico, Antman [12] reported that the migration of adult children was associated with poorer physical and mental health outcomes for ageing parents. Studies conducted among the older parents in general also show that close contact and emotional cohesion with children is associated with improved parental mental health. For instance, Dykstra and de Jong Gierveld [13] found that social and emotional loneliness among older Dutch women was negatively associated with weekly contact with their children. Similarly, older European parents who saw or talked to their children more often than once a week had significantly lower levels of depression [14]. Among the Chinese elderly, living alone was associated with low subjective well-being and living with immediate family members improved their general well-being [15]. Internal migration of children in Indonesia had a negative effect on elderly parents’ daily living, self-rated health and mortality [16].

In contrast, there are studies reporting better physical and emotional well-being among the left behind elderly parents. Waite and Hughes [17] found that left behind parents in the USA enjoyed improved health conditions over parents living with their children. A study in China [18] reported non-empty nest elderly utilizing better health care than that of empty nest elderly. Wenger et al. [19] in their multi-country study showed that elders whose children were living away had more freedom with more time to make friends, and engage in social activities. Living alone provides parents with an opportunity for reconnection and reawakened interests [20, 21]. In Moldova [22], better physical health among the left behind elderly parents was a consequence of their children’s migration. However, this study and a similar study by Gibson et al. [23] in Tonga showed no effect of the migration on the mental health of parents.

Among the left behind, a number of risk factors for poorer mental health have been identified ranging from predisposing inherent factors (such as age, sex, education, existing disease status, previous mental illness, and place of residence) to a wider community and social factors such as existing social support, number of social ties, community engagement and interactions, and access to health services. In general, males, younger parents, living in urban areas, and better access to medical care are positively associated with improved mental health of empty nesters. Despite the increased focus of research in this area, the empirical findings are equivocal. Research on the consequences of children’s migration on the mental health and well-being of elderly people remains inconclusive and a systematic review is warranted.

### Objective of the review

To identify the association between the left behind or empty nest status and the mental health of elderly parents and to identify the common risk factors for poor mental health among those left behind.

## Materials and methods

This integrative review considered research relating to the migration of children and the mental health of the left behind parents. Integrative reviews are an effective method for combining studies with diverse methodologies and data sources in order to increase understanding of the topic, subsequently contributing to the evidence-base [24].

### Studies identification

Well-established databases (CINAHL, PsycINFO, PubMed, Scopus and ProQuest) were searched for research published in English language to identify relevant studies on mental health status of left behind parents or elderly people. The following search terms were used: ‘left behind’; ‘country staying’; ‘left in hometown’; ‘left in rural areas’; ‘stay at home’; ‘empty nest’; ‘empty nester’; parents; elderly; aged; adult; aging; ‘mental health’; ‘mental disorders’; ‘psychological well-being’; ‘well-being’; and ‘quality of life’. The search strategy was supplemented by review of the reference lists of the included research [25].

### Inclusion and exclusion criteria

Included studies met the following criteria: (a) focused on the relationship between the migration of the adult child(ren) and the mental health of the elderly parents (≥50 years) left behind or factors related to the mental health of the left behind parents; and (b) published in English from January 2000 to September 2017.

Studies were excluded if the focus was on the left behind children, spouse or family members. In addition, studies related to parents and/or elderly left behind due to the death of a child were excluded. To account for the cohort effect, studies published before the year 2000 were excluded.

**Fig 1:**
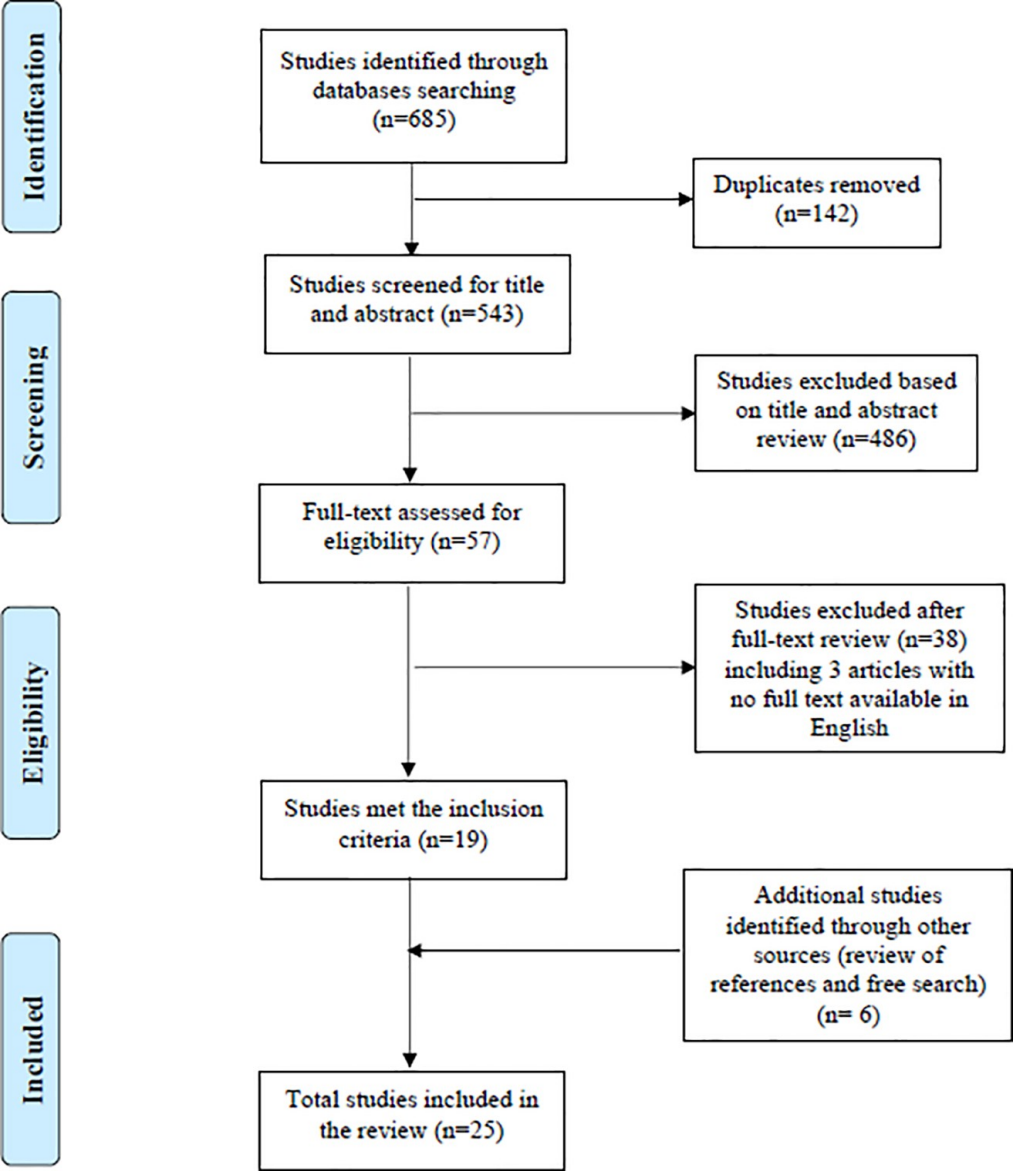
Study selection process for the review.

The process of selection included reviewing the titles and abstracts to identify potential articles and then reading the full text to determine whether articles met the inclusion criteria. Initial screening was carried out by the first author and then checked independently by all other authors. The final sample comprised 25 articles from 23 studies that met the inclusion criteria (see Fig 1).

### Data abstraction

The first author (DKT) extracted and coded the following information: authors’ names, publication year, country, design, purpose, sample size, age of participants, mental health related variable(s), data collection tools/scales and data analysis method (Table 1), prevalence and/or mean scores of the scales in left behind and non-left behind groups (Table 2), and factors associated with mental health among the left behind group (Table 3). The other authors (DV RK and MC) verified the extracted data. The variety of tools and instruments used to assess mental health precluded a quantitative meta-analysis.

**Table 1:** Summary of the included studies.

SN	Study, year and country	Design	Purpose	Sample and study population	Mental health related variable(s)	Data collection method/tools (cut-offs) Scale reference	Data analysis
1	Gao et al. 2017 [26] China	Longitudinal	*To analyse the relationship between an empty nest and the overall health of the elderly*, *and explored the mechanisms behind how an empty nest influences the health of the elderly in urban and rural China* (p. 3)	7823, ≥65 years (3297 EN & 4526 non-EN)	Cognitive ability	MMSE (-) [27]	Regression analysis
					Psychological health	Researcher developed scale (-)	
2	Waidler et al. 2017 [28] Moldova	Cross sectional	*To evaluate the wellbeing of elderly individuals ‘left behind’ by their adult migrant children in Moldova* (p. 607)	1322, ≥60 years (505 LB & 817 Non-LB)	Depression	MHI-38 (≥13) [29]	Regression analysis
3	Mosca and Barrett 2016 [30] Ireland	Longitudinal	*To explore whether older parents of adult children who emigrate experience*, *in the short term*, *increases in depressive symptoms and loneliness feelings compared to parents whose children do not migrate*. (p. 687)	2523, ≥50 years (357 LB & 2166 Non-LB)	Depression	CES-D (≥16) [31]	Regression analysis
					Loneliness	UCLA-LS (-) [32]	
4	Guo et al. 2016 [33] China	Cross sectional	*To compare mental health and related influencing factors among the empty-nest and the non-empty-nest elderly*. (p. 210)	488, ≥60 years (268 EN & 220 non-EN)	Abnormal mental symptoms	SCL-90-R (-) [34]	Regression analysis
5	Downer et al. 2016 [35] Mexico	Longitudinal	*To examine if older adults in Mexico who have one or more adult children living in the United States are more or less likely to develop cognitive impairment over an 11-year period compared to older adults who do not have any adult children living in the United States*. (p. 1)	2609, (673 LB & 1936 Non-LB)	Baseline: Cognitive impairment	CCCE [36]	Logistic regression
					Follow up: Cognitive impairment	IQCODE (abbreviated version) [37]	
	Antman 2010 [12] Mexico	Cross sectional	*To explore whether elderly parents of children in the U*.*S*. *suffer from worse health outcomes than their counterparts with no children in the U*.*S*. (p. 205)	6730, ≥60 years (1483 LB & 5247 Non-LB)	Mental health	Self-reported mental health (-)	Regression analysis
6	Chang et al. 2016 [38] China	Cross sectional	*To comprehensively compare the general characteristics*, *lifestyles*, *serum parameters*, *ultrasonic cardiogram parameters*, *depression*, *quality of life*, *and various comorbidities between empty nest and non-empty nest elderly* (p. 2)	3208, ≥60 years (1669 EN living as a couple, 271 EN living alone & 1268 non-EN)	Depression	PHQ-9 (≥5) [39]	Logistic regression
					Psychological dimension of WHOQOL-BREF	WHOQOL-BREF (-) [40]	
7	He et al. 2016 [41] China	Cross sectional	*To investigated the prevalence of depression and the associated factors that influence depression in the left-behind elderly population in a rural area of China* (p. 638)	509 LB, ≥65 years	Depression	GDS-30 (≥11) [42]	Multiple linear regression
8	Böhme et al. 2015 [22] Moldova	Cross sectional	*To investigate the effect of migration on various dimensions of elderly health using unique data from Moldova*. (p. 211)	1566, ≥60 years (925 LB & 614 Non-LB)	Mental health	MHI-5 (-) [29]	Regression analysis
9	Zhai et al. 2015 [43] China	Cross sectional	*To investigate the association of empty nest with depressive symptom in a Chinese elderly population*. (p. 218)	9215, ≥60 years (5289 EN & 3926 Non-EN)	Depression	PHQ-9 (≥5) [39]	Logistic regression
					Cognitive impairment	MMSE (<24) [44]	
10	Cheng et al. 2015 [45] China	Cross sectional	*To determine the disparities in prevalence and risk factors of loneliness between rural empty nest and non-empty nest older adults*. (p. 356)	730, ≥60 years (381 EN & 349 non-EN)	Loneliness	UCLA-LS (-) [32]	Pearson’s correlation, Multivariate linear regression
					Depression	GDS-30 (≥11) [42]	
					Psychological dimension of WHOQOL-BREF	WHOQOL-BREF (-) [40]	
11	Liang and Wu 2014 [46] China	Cross sectional	*To explore the health-related quality of life of empty-nest elderly in rural China* (p. 1)	967 EN, ≥60 years	Anxiety/depression	EQ-5D (-) [47]	Regression analysis
12	Xie et al. 2014 [48] China	Cross sectional	*To investigate the quality of life and the associated factors on left behind elderly in rural China* (p. 364)	434 LB, ≥60 years	Psychological health	WHOQOL-BREF Chinese version (-) [40]	Multiple linear regression
13	Sekhon and Minhas 2014 [49] India	Cross sectional	*To get an insight into the mental health of the elderly people* (p. 31)	620,≥60 years from families which had at least one member permanently emigrated abroad	Depression	Self-reported depression (Yes/No)	Descriptive
14	Wang et al. 2013 [50] China	Cross sectional	*To determine the prevalence and correlates of anxiety disorders among empty-nest older adults in Sichuan Province*, *China* (p. 298)	352,≥60 years who were not living with any children	Anxiety disorders	SAS (SAS standard score ≥50) [51]	Stepwise multivariable regression
					Depression	GDS-15 (-) [52]	
					Loneliness	UCLA-LS (-) [32]	
					Cognitive impairment	MMSE (<24) [44]	
15	Abas et al. 2013 [53] Thialand	Longitudinal	*To test for prospective associations between (1) out-migration of all children and subsequent depression in parents and (2) having a child move back and an improvement in parents’ depression*. (p. 226)	960,≥60 years (all the children migrated 805& at least one child inside district 155)	Depression	EURO-D (>12) [54]	Logistic regression
16	Su et al. 2012 [55] China	Cross sectional	*To compare levels of depression and social support among empty-nest elderly who living in the rural and urban area of Hunan province*, *China* (p. 564)	809 EN,≥60 years	Depression	GDS-30 (≥11) [42]	Two level linear mixed-effects model
17	Adhikari et al. 2011 [56] Thialand	Cross sectional	*To explore the impact of migration on the health of the elderly left behind and their health care-seeking behavior*. (p. 2)	28677,≥60 years (19275 LB & 9402 Non-LB)	Symptoms of poor mental health	Research developed composite indicator (-)	Logistic regression
18	Sun et al. 2011 [57] China	Cross sectional	*To compare health-related quality of life for elderly men and women in three mutually exclusive living arrangements*: *living alone*, *living only with spouse*, *and non-empty-nesters*. (p. 359)	9711, ≥60 years (-)	Anxiety/Depression	EQ-5D (-) [47]	Logistic regression
19	Xie et al. 2010 [58] China	Cross sectional	*To clarify the prevalence of depression among empty-nest elderly and evaluate the impact of social support*, *coping style and socio-demographic factors on depression of the empty-nest elderly* (p. 25)	414, ≥60 years (230 EN & 184 non-EN)	Depression	GDS-30 (≥11) [42]	Multiple linear regressions
20	Abas et al. 2009 [59] Thialand	Cross sectional	*To describe correlates of outmigration and to estimate any association between outmigration of children and depression in rural-dwelling older parents*. (p. 54)	1147, ≥60 years (182 all children out, 78 some children out & 187 no children living out)	Depression	EURO-D (-) [54]	Regression analysis
21	Liu and Guo 2008 [60] China	Cross sectional	*To estimate the life satisfaction and its predictors between the empty-nest and not empty nest elderly*. (p. 823)	590, ≥60 years (275 EN & 315 non-EN)	Depression	GDS-30 (-) [42]	Multiple linear regression
					Life satisfaction	LSI (-) [61]	
	Liu and Guo 2007 [62] China		*To estimate whether loneliness was associated with quality of life and examined the influence of socioeconomic factors in the empty nest elderly*. (p. 1275)		Loneliness	UCLA-LS (-) [32]	
					Mental health	SF-36 (-) [63]	
22	Liu et al. 2007 [18] China	Cross sectional but reported as case-control	*(i) To compare health-care utilization and perceived unmet needs between elderly empty-nesters in rural areas and those in cities to identify if the rural empty-nesters have equitable access to health services*. *(ii) To compare the factors associated with health-care utilization between the two groups*. (p. 407)	490, ≥60 years (250 EN & 240 non-EN)	Mental health	SF-36 (-) [63]	t-test, chi-square test and principal component analysis
23	Miltiades 2002 [64] India	Qualitative	*To examine the effect an adult child’s emigration has on the familial support system available to the parents left behind*, *and on the parent’s psychological well-being*. (p. 33)	29 parents (≥60 years) who had adult children in the United States	Psychological well-being	-	Grouping, coding, comparing and contrasting (context and thematic analysis)

**Abbreviations:** EN: Empty nest, MMSE: Mini-Mental State Examination, LB: Left behind, MHI: Mental health inventory, CES-D: Center for Epidemiologic Studies Depression Scale, UCLA-LS: University of California Los Angeles Loneliness Scale, SCL-90-R: Symptom Checklist-90-Revised, CCCE: Cross-Cultural Cognitive Examination, IQCODE: Informant Questionnaire on Cognitive Decline in the Elderly, PHQ-9: Patient Health Questionnaire-9 scale, WHOQOL-BREF: World Health Organization Quality of Life Questionnaire abbreviated version, GDS: Geriatric Depression Scale, EQ-5D: European Quality of Life-5 Dimensions, SAS: Self Rating Anxiety Scale, EQ-12: European Quality of Health Scale, EURO-D: European Version of Depression Scale, LSI: Life Satisfaction Index, SF-36: 36-Item Short-Form Health Survey.

‘-’ indicates not available or not reported.

**Table 2:** Prevalence and mean scores of mental health measures.

SN	Study	Age (years)		Aspects of mental health	Scale/instrument (Cut off)	Left behind parents		Non-left behind parents		Significance
		Inclusion criteria	Mean±SD			N	Prevalence % or Mean score±SD or Both	N	Prevalence % or Mean score±SD or Both	
1	Gao et al. [26]^1^ China	≥65	LB : 79.6 & Non-LB : 84.9	Cognitive ability	MMSE (-)	3297	18.9±5.5	4526	14.8±14.9	***(p<0.001)
				Psychological health	Researcher developed scale (-)		17.7±10.4		14.6±13.9	***(p<0.001)
2	Waidler et al. [28] Moldova	≥60	_	Depression	MHI-38 (≥13)	505	28.7%	817	29.0%	NS
3	Mosca and Barrett [30] Ireland	≥50	LB: 60.3±5.1 & Non-LB: 62.9±6.4	Depression	CES-D (≥16)	357	4.7±6.0	2166	6.1±7.8	***
				Loneliness	UCLA-LS (-)		1.5±1.9		1.8±2.1	**_
4	Guo et al. [33] China	≥60	69.9±7.6	Abnormal mental symptoms	SCL-90-R	268	11.9%	220	11.8%	_
5	Downer et al. [35] Mexico	≥60	LB: 66.2±5.3 & Non-LB: 66.6±5.5	Cognitive impairment	IQCODE	673	15.3%	1936	16.3%	NS(p = 0.54)
	Antman [12] Mexico	_	LB: 62.9±8.9 & Non-LB: 61.3±9.4	Poor mental health	Researcher developed measure (-)	1483	0.6±0.4	5247	0.5±0.7	***(p<0.001)
6	Chang et al. [38] China	≥60	67.0±5.8	Depression	PHQ-9 (≥5)	271 living alone & 1669 living as a couple	26.9% (3.6±4.5) & 24.7% (3.1±3.8)	1268	26.9% (3.3±3.9)	NS
				Psychological health	WHOQOL-BREF		14.4±2.3 & 14.4±2.5		14.4±2.5	
7	He et al. [41] China	≥65	_	Depression	GDS-30 (≥11)	509	36.9%			
8	Böhme et al. [22] Moldova	≥60	69.3	Mental health	MHI-5 (-)	614	18.5	925	18.6	NS
9	Zhai et al. [43] China	≥60	Median: 68.0	Depression	PHQ-9 (≥5)	5289	11.6%	3926	8.6%	***(p<0.001)
				Cognitive impairment	MMSE (<24)	5452	15.7%	3926	13.2%	**(p = 0.001)
10	Cheng et al. [45] China	≥60	LB: 69.1 & Non-LB: 68.1	Depression	GDS-30 (≥11)	381	28.6% (7.7±6.4)	349	24.1% (6.8±5.9)	*(p = 0.043)
				Loneliness	UCLA-LS (-)		41.5±7.0		39.5±7.4	***(p<0.001)
				Psychological health	WHOQOL-BREF (-)		13.5±1.9		13.8±1.9	*(p = 0.011)
11	Liang and Wu [46] China	≥60	78.3±9.6	Anxiety/depression	EQ-5D	958	82.0%			
12	Xie et al. [48] China	≥60	_	Psychological domain of quality of life	WHOQOL-BREF (-)	434	39.6±13.7	Population	61.6±13.7	***(p<0.001)
13	Sekhon and Minhas [49] India	≥60	_	Depression	Self-reported depression	620	98.0%			
14	Wang et al. [50] China	≥60	69.1±7.1	Anxiety	SAS (SAS standard score ≥50)	352	30.1% (44.5±11.0)			
				Depression	GDS-SF (-)		3.7±3.1			
				Loneliness	UCLA-LS (-)		35.6±9.9			
				Cognitive impairment	MMSE (<24)		22.1±6.8			
15	Abas et al. [53] Thialand	≥60	69.0±6.7	Depression	EURO-D (>12)	All the children migrated:155	16.0%	At least one child inside district: 805	27.0%	**(p = 0.001)
16	Su et al. [55] China	≥60	70.1±7.9	Depression	GDS-30 (≥11)	809	73.3% (14.0±5.9)			
17	Adhikari et al. [56] Thialand	≥60	_	Symptoms of poor mental health	Researcher developed measure (-)	19275	58.9%	9402	56.0%	_
18	Sun et al. [57] China	≥60	_	Anxiety/Depression	EQ-5D	-	-	-	-	
19	Xie et al. [58] China	≥60	70.2±7.9	Depression	GDS-30 (≥11)	231	79.7%	184	67.9%	**(p = 0.003)
20	Abas et al. [59] Thialand	≥60	69.8±7.1	Depression	EURO-D (-)	All children migrated: 182	2.9	No children migrated: 187	3.7	**(p = 0.001)
						Some children migrated: 778	4.0			
21	Liu and Guo [60] China	≥60	EN: 69.8±6.7 & Non-EN: 69.9±8.7	Depression	GDS-30 (-)	275	8.8±6.5	315	7.7 ±6.1	*(p = 0.028)
				Loneliness	UCLA-LS (-)		35.9±9.4		34.1 ± 9.3	* (p = 0.017)
				Life satisfaction	LSI (-)		18.1±6.1		19.5 ±5.2	**(p = 0.003)
	Liu and Guo [62] China			Mental health subscale	SF-36 (-)		69.1±18.3		72.8±15.1	*(p = 0.010)
22	Liu et al. [18] China	≥60	EN: 69.5±6.1 & Non-EN: 70.3±9.7	Mental health subscale	SF-36 (-)	250	68.6±17.7	240	77.4±20.3	** (p<0.01)
23	Miltiades [64] India	≥50	NA	Not clear		29	Emigration places a heavy psychological burden on the parents.			

^1^The study reported the scores after logarithmic transformation. We report the raw scores.

*p<0.05.

**p<0.01.

***p<0.001.

–not available/not reported.

**Table 3:** Factors related to mental health among 'left behind' older people.

SN	Studies	Association (LB/EN and mental health)			Factors related to mental health among the left behind older people								
		Mental health aspects measured	Effect measure (95% CI)	P value	Left behind/ Empty nest	Sex	Age	Marital status/ residence type	Place of residence	Education	Income	Physical health	Other
1	Gao et al. [26] China	Cognitive ability (MMSE score)	β: (Urban) -3.585 (-)	<0.001	↓	Male↑	↓	Married↑	_	_	_	_	Exercise↑
		Cognitive ability (MMSE score)	β (Rural): -2.438 (-)	<0.001									
		Psychological health	β: (Urban) -3.751 (-)	<0.001									
		Psychological health	β (Rural): -2.595 (-)	<0.001									
2	Waidler et al. [28] Moldova	Depression (MHI-38) (Not depressed)	β: 0.31 (-)	NS	=	_	_	_	_	_	_	_	_
3	Mosca and Barrett [30] Ireland	Depression (CES-D)	β: 0.0575 (-)	< 0.05	↓	_	_	_	_	_	_	_	_
4	Guo et al. [33] China	Abnormal mental symptoms	Δp = 0.001^1^	-	= ^1^	Male↑	↓	=	Urban = Rural	=	↑	Chronic disease↓	_
5	Downer et al. [35] Mexico	Cognitive impairment	OR: 0.86 (0.61–1.21)	NS	=	_	_	_	_	_	_	_	_
		Depression	OR: 1.96 (1.24–3.04)	<0.05	↓								
	Antman [12] Mexico	Poor mental health	β: 0.082	0.041	↓	Male↑	_	_	_	↑	_	_	_
6	Chang et al. [38] China	Depression	OR: 0.94 (0.79–1.11)^ and 1.03 (0.76–1.40)⁺	NS	=	_	_	_	_	_	_	_	_
7	He et al. [41] China	Depression	-	-	-	Male↑^1^	↓^1^	Living with spouse↑^1^	_	↑^1^	Financial support↑^1^	Two or more chronic disease↓^1^	Physical activity↑^1^, frequency of children's visit↑^1^
8	Böhme et al. [22] Moldova	MHI-5	β: −0.07	NS	=	_	_	_	_	_	_	_	_
9	Zhai et al. [43] China	Depression	OR: 1.22 (1.05–1.43)	0.012	↓	_	_	_	_	_	_	_	_
10	Cheng et al. [45] China	Loneliness	$\Delta \bar{x}$ = 2.06	<0.001^1^	↓	=	↑	Married↑^1^	_	↑^1^	Income↑^1^, Farmers↓^1^	Any chronic disease = ^1^	Family support↑, Social interaction↑, General quality of life↑, Poor sleep quality↓^1^, Smoking = , Drinking =
11	Liang and Wu [46] China	Anxiety/Depression (EQ-5D)	-	-	-	=	↑	Widowed = non widowed	_	↑	_	_	_
12	Xie et al. [48] China	Psychological health (WHOQoL-BREF)	-	-	-	Male↓^1^	_	Living with spouse↑^1^	_	_	_	_	Frequency of children's visit↑^1^
13	Sekhon and Minhas [49] India	Depression	-	-	-	_	_	_	_	_	_	_	_
14	Wang et al. [50] China	Anxiety (SAS)	-	-	-	Male↑^1^	= ^1^	Living with spouse↑	Urban↑	↑^1^	Income↑^1^, Skilled worker↑^1^	_	
15	Abas et al. [53] Thialand	Depression (EURO-D)	OR: 0.46 (0.210–0.985)	0.046	↑	_	_	_	_	_	_	_	_
16	Su et al. [55] China	Depression (GDS-30)	-	-	-	_	_	_	Urban↑^1^	_	Self-perceived income↑	Body disease↓	Physical activity↑
17	Adhikari et al. [56] Thialand	Symptoms of poor mental health	OR: 1.10 (1.05–1.17)	<0.001	↓	_	_	_	_	_	_	_	_
18	Sun et al. [57] China	Anxiety/Depression (EQ-5D)	OR: 1.10 (0.94–1.30)^ and 1.73 (1.41–2.13)⁺	NS in case of ^ and <0.01 in case of ⁺	↓	_	_	_	_	_	_	_	_
19	Xie et al. [58] China	Depression (GDS)	Δp = 0.118	0.003^1^	↓	__	__	Married↑	__	__	Self-perceived income↑	__	Social support↑, religious belief↓, positive coping↑
20	Abas et al. [59] Thialand	Depression (EURO-D)	β: 0.91 (0.26–1.57)^2^ and 1.05 (0.35–1.75)^3^	0.013	↑	_	_	_	_	_	_	_	_
21	Liu and Guo [60] China	Life satisfaction (LSI-Z)	β: -0.606	<0.001	↓	_	↓^1^	Married↑	_	↓	↑	Any chronic disease↓	Social support↑, Relationship with children (good)↑
	Liu and Guo [62] China	Loneliness	$\Delta \bar{x}$ = 1.85	0.017^1^									
22	Liu et al. [18] China	Mental health	$\Delta \bar{x}$ = -8.74	<0.01	↓	_	_	_	_	_	_	_	_
23	Miltiades [64] India	-	-	-	-	_	_	_	_	_	_	_	_

β: Regression coefficient

OR: Odds Ratio

↑ increase factor is associated with improved mental health (positive association with positive aspect of mental health)

↓ increase factor is associated with decreased mental health (negative association with positive aspect of mental health)

= no difference (no significant association)

–refers to ‘not given’

^ Living as a couple

⁺ Living alone

$\Delta \bar{x}$ mean difference

Δp proportion difference

^1^Bivariate association

^2^some children migrated vs. all children migrated

^3^no children migrated vs. all children migrated.

**Note:** Only variables predicting significant association in multivariate analysis are included in the table, otherwise indicated.

### Quality assessment

The JBI Checklist for Analytical Cross Sectional Studies [65] was adapted to assess quality. Articles were scored *Yes*, *No*, *Unclear* or *Not Applicable* (NA) for the following: (1) criteria for inclusion in the sample clearly defined, (2) study subjects and the setting described in detail, (3) exposure measured in a valid and reliable way, (4) objective, standard criteria used for measurement of the condition, (5) confounding factors identified, (6) strategies to deal with confounding factors stated, (7) outcomes measured in a valid and reliable way, and (8) appropriate statistical analysis used. (See S1 Table)

## Results of the review

### Study characteristics

Twenty-three studies reported in 25 publications from six different countries were included. The majority were from China (n = 14). Other countries included Thailand (n = 3), Moldova and India (n = 2), and Mexico and Ireland (n = 1). Four studies were longitudinal [26, 30, 35, 53] with the remainder cross sectional with the exception of one qualitative study [64].

The majority of studies (n = 14) used random sampling [18, 22, 33, 38, 41, 43, 45, 46, 50, 53, 55–60, 62] while five did not provide sampling information [12, 26, 28, 30, 48]. One used total sampling [49] and another used snowball sampling [64]. The sample size of quantitative studies ranged from 352 to 28,677, and the qualitative study had 29 participants. The age of subjects ranged from 50 to 100 plus years.

Nine studies [26, 33, 38, 41, 45, 48, 55, 58, 60, 62] reported a response rate above 90% while five [18, 30, 46, 53, 59] had a response rate range of 80–90%. The remaining eight [12, 22, 28, 43, 49, 50, 56, 57, 64] did not report the response rate.

### Defining ‘left behind’ and ‘empty nest’

Thirteen articles were related to ‘empty nest’ [18, 26, 33, 38, 43, 45, 46, 50, 55, 57, 58, 60, 62] while the remaining 12 discussed the ‘left behind’ [12, 22, 28, 30, 35, 41, 48, 49, 53, 56, 59, 64]. There was uniformity on the use of the term ‘empty nest’, as elders who living alone, or with a spouse only, were defined as empty nest and those living with family members were considered non-empty nest across all studies. The elderly without children were deemed empty nest if living alone or with a spouse, however one study [43] excluded elderly who were childless. For the studies reporting ‘left behind’, the inclusion criteria included elderly parents having (adult) children and at least one of the children having migrated—excluding those without any living child. There were some variations in defining the duration of migration. He et al. [41], for example, defined ‘left behind elderly’ as those with adult children having left for more than 6 months while two studies [22, 53] defined migrant children having left home for more than 3 months. Antman [12] and Downer et al. [35] defined parents as left behind if any of their children were living in the USA. Sekhon and Minhas [49] considered families which had at least one member who had permanently emigrated abroad. A follow up study defined left behind as no children emigrated at baseline but one or more children emigrated at follow up [30]. Xie et al. [48] did not provide clear defining criteria.

Sixteen studies [12, 18, 22, 26, 28, 30, 33, 38, 43, 45, 53, 56–60, 62] had a control group. Two studies [38, 57] separated the left behind group into ‘living alone’ and ‘living with spouse/as couple’. Abas et al. [59] compared the mental health status across three groups: ‘all children migrated’, ‘some children migrated’ and ‘no children migrated’. The remaining seven publications [41, 46, 48–50, 55, 64] studied the left behind and did not have a comparison group. Sixteen studies [12, 26, 28, 30, 33, 41, 43, 45, 46, 48, 50, 55, 57, 58, 60, 62] were concerned with factors affecting the mental health status of the left behind, while the remaining eight only assessed the relationship between the children’s migration and the mental health status of parents.

### Measures of mental health

A range of measures were used to assess mental health status with many using multiple measures. Depression was assessed in 13 studies [28, 30, 38, 41, 43, 45, 49, 53, 55, 58–60] and three studies assessed loneliness [30, 45, 62]. Other measures of mental health included anxiety [50], cognitive function [43, 50], life satisfaction [60] and social isolation [62]. Some used broader measures such as symptoms of poor mental health [56], self-reported mental health [12, 30], psychological well-being [64], psychological health [26, 48] and measures of mental health status [18, 22, 33].

Twenty studies used standard instruments for measuring different aspects of mental health. Depression was measured by the Center for Epidemiologic Studies Depression Scale (CES-D) [30], Geriatric Depression Scale (GDS) [41, 45, 50, 55, 58, 60, 62], European Quality of Life-5 Dimensions (EQ-5D) [46, 57] and the European Version of Depression Scale (EURO-D) [53, 59]. Cognitive function of the elderly was assessed using the Mini-Mental State Examination (MMSE) [26, 43], Cross-Cultural Cognitive Examination (CCCE) and the Informant Questionnaire on Cognitive Decline in the Elderly (IQCODE) [35]. Instruments used to measure mental health included the Mental Health Inventory (MHI) [22, 28], University of California Los Angeles Loneliness Scale (UCLA-LS) [30, 45, 60, 62], Symptom Checklist-90-Revised (SCL-90-R) [33], Patient Health Questionnaire-9 scale (PHQ-9) [38, 43], Self-Rating Anxiety Scale (SAS) [50], Short Form Health Survey (SF) [18, 62] and the Life Satisfaction Index (LSI) [60, 62]. Three studies [38, 45, 48] used the World Health Organization Quality of Life Questionnaire abbreviated version (WHO-BREF).

### Mental health of the ‘left behind’ parents

#### Depression

Thirteen studies reported depression among the left behind elderly parents. All studies used validated scales to measure depression, except Sekhon and Minhas [49] who asked *‘Do you feel depressed that your family member has gone abroad and is no longer staying with you*?*’* with 98% of participants responding *‘Yes’*. Among the studies that used validated scales, an equal number (n = 4) reported both prevalence and mean scores, prevalence only and mean scores only. Studies using cut-off scores (n = 8) reported the prevalence of depression among the left behind elderly ranging from 11.6% to 79.7%. Large variations in the mean score of depression were observed (Table 2).

Variation in scales resulted in large heterogeneity in depression prevalence as well as mean scores. Among the seven different scales, the Geriatric Depression Scale-Long Form (GDS-30) [42] was the most commonly used. The GDS-30 consists of 30 items with a score ranging from 0 to 30, higher scores represent increased depression. GDS scores of 11 and above suggest depressive symptoms. The studies using GDS-30 reported the proportion of left behind elderly having depressive symptoms ranging from 36.9% to 79.7% and mean GDS score from 7.7 and 14.0. Wang et al. [50] used GDS-Short form [52] comprising 15 items with a score range from 0 to 15 and reported a mean score of 3.7.

Two studies applied EURO-D, a 12-item depression screening scale with a cut-off of 6 [54]. Abas et al. [53] used a cut-off core of 12 and reported a prevalence of 16% among the elderly with all children migrated. Abas et al. [59] reported mean scores of 2.9 for the elderly with all children migrated and 4.0 for some children migrated. Two studies used the Patient Health Questionnaire (PHQ-9) with 9 items, with a scoring range of 0 to 27 [39]. Zhai et al. [43] used a cut-off of 5 and found a depression prevalence of 11.6%. Chang et al. [38] used two different cut-offs (5 and 10), resulting in the reported prevalence of depression of 26.9% and 8.1% for elderly living alone and 24.7% and 5.9% for elderly living as couple. A study using the Mental Health Inventory (MHI-38) [29] reported a mean score of 71.3 [28]. Similarly, a study using SCL-90-R [34] reported depression among 13.1% of the left behind elderly with a mean depression score of 1.5 [33].

Mosca and Barrett [30] used the 20-item Center for Epidemiologic Studies Depression Scale (CES-D) [31] to measure depressive symptoms in the week prior to the interview. Each of the 20 items was scored on a four-point scale leading to a total score of 60, with higher scores indicating higher depressive symptoms, with a mean depression score of 4.7.

Two studies used the EQ-5D scale developed by The EuroQol Group [47] to measure health-related quality of life among the empty nest elderly in rural China. Liang and Wu [46] reported an anxiety/depression prevalence of 82% using EQ-5D while Sun et al. [57] reported the depression prevalence only for sex and age sub groups.

#### Anxiety

Wang et al. [50] determined the prevalence of anxiety disorders using the Self-Rating Anxiety Scale (SAS) [51]. The SAS is a 20-item scale with scores ranging from 20 to 80, with higher scores representing higher anxiety. A cut-off of 50 was used for a SAS standardized score = 1.5 x SAS sum score. The mean standardized score of 44.5 indicated relatively low anxiety, while the prevalence of anxiety disorders was 30.1%. The mean SAS standardized scores were higher in females (46.7) compared to males (42.5); elderly living alone (46.3) compared to living with spouse (43.9); rural inhabitants (48.9) compared to urban (39.7); and unmarried/single/divorced or widowed (48.1) compared to married (43.8). In addition, the study also reported the association of anxiety with education level, occupation and monthly income of the elderly.

#### Cognitive impairment

Cognitive function of the left behind elderly was assessed using the Mini Mental State Examination (MMSE), a 30-item test to assess orientation, attention, calculation, language, and recall [44]. The MMSE yields a score of 0–30 (cut-off of 24) with higher scores indicating better functioning. Zhai et al. [43] found 15.7% of the elderly with cognitive impairment while Wang et al. [50] reported a mean MMSE score of 22.1 (SD = 6.8). The Chinese version of the MMSE [27] with 25 items (score 0 to 25) was used by Gao et al. [26] reporting a mean score of 18.9 (SD = 5.5).

#### Loneliness

Loneliness was assessed using the University of California, Los Angeles Loneliness Scale (UCLA-LS) [32] which consists of 20 questions, using a four-point scale, with a total score range of 20 to 80 with higher scores indicating increased loneliness. The mean UCLA-LS scores reported were 35.7 (SD = 9.9) [50], 41.5 (SD = 7.0) [45] and 35.9 (SD = 9.4) [62]. UCLA scores of 20–34, 35–49, 50–64 and 65–80 are considered to be mild, moderate, moderate–severe, and severe loneliness, respectively [66]. Cheng et al. [45] reported the prevalence of mild, moderate, moderate–severe and severe loneliness as 14.4%, 75.8%, 9.9%, and 0% respectively. Similarly, Liu and Guo [62] found 45.5% experiencing mild loneliness, 43.6% moderate, and 10.9% moderate–severe loneliness and no severe loneliness. Mosca and Barrett [30] included only five items of ULCA-LS and reported a mean score of 1.5 (in a range of 0 to 10).

#### Other general measures of mental health

The World Health Organization Quality of Life Questionnaire abbreviated version (WHOQOL-BREF) [40] consists of 26 items containing two objective items (overall QOL and general health status) and 24 other items divided into four domains: physiological (seven items), psychological (six items), social relationships (three items) and environment (eight items). Each item is scored from 1 to 5 and domain scores range from 4 to 20 points (mean score for all items × 4) with a higher score representing better quality of life. In this review, only scores for the psychological domain are relevant. Cheng et al. [45] report a mean score of 13.5 (SD = 1.9), and Xie et al. [48] converted the score into a centesimal grade [(original score– 4) × (100/16)] and reported the mean score of 39.6 (SD = 13.8). The equivalent centesimal score for the Cheng et al. [45] is 59.1.

Böhme et al. [22] assessed the psychological well-being of elderly parents with at least one biological child staying abroad for at least three months during the year prior to the survey. The study used MHI-5, a five question scale based on Mental Health Inventory developed by Veit and Ware [29] ranging from 5 (very poor) to 30 (very good). The mean score of psychological well-being reported by Böhme et al. [22] was 18.5.

The 36-Item Short-Form Health Survey (SF-36) [63] was used to assess general health with Mental Component Summary (MCS) scores ranging from 0 to 100, where higher scores indicate better mental health. Studies reported mean scores of 69.1 (SD = 18.3) [62] and 68.6 (SD = 17.7) [18].

Antman [12] created the ‘Poor Mental Health’ variable, equal to 1 if the respondent reported feeling depressed, lonely, or sad for the week prior to the survey otherwise 0. The mean score of ‘Poor Mental Health’ was 0.6 (SD = 0.01) among the left behind parents. Adhikari et al. [56] using their own instrument reported 58.9% of the left behind elderly having symptoms of poor mental health.

### Children’s migration status and mental health of left behind parents

Among the studies that compared prevalence or mean scores between the left behind and non-left behind elderly parents (n = 15), ten reported statistically significant differences while three were non-significant. Two studies [33, 56] did not provide details on significance. Nine studies found the mental health status of the left behind elderly to be poorer than that of the elderly parents living with their children with statistically significant differences in six studies. More specifically, these studies showed that left behind parents had higher depressive symptoms [43, 45, 58, 60], higher levels of loneliness [45, 60], lower life satisfaction [60], lower cognitive ability [43] and poorer psychological health [12, 18, 33, 45, 56, 62].

Three studies found statistically significant differences showing better mental health among the left behind, with one further study showing a non-significant difference. Gao et al. [26] reported higher cognitive ability and improved psychological health scores among the left behind, however confounding by age may account for this result. Decreased prevalence of depression among the left behind parents was reported [28, 30, 53]. Two studies classified left behind into two groups, among which Chang et al. [38] found a lower proportion of depression among the elderly living with a spouse. Similarly, Abas et al. [53] reported the highest mean depression scores among the elderly with ‘some children living outside’, followed by ‘no children living outside’ and ‘all children living outside’ (Table 3). Guo et al. [33] stated that the mental health status of the left behind parents was better than that of the non-left behind but reported similar results for both groups.

Sixteen studies analysed the association between the left behind and the mental health of elderly, of which 12 studies conducted multivariate analysis and the remaining four studies reported only bivariate association. For multivariate analyses, seven studies [12, 26, 30, 43, 56, 57, 60] showed that parents whose children had migrated were at greater risk of mental health problems than those with non-migrant children (Table 3). For instance, Gao et al. [26] found a negative association of empty nest with cognitive ability and psychological health in both urban and rural elders. Depressive symptoms were found to be higher among the parents of migrant children [30, 43]. Sun et al. [57] reported that the risk of anxiety/depression was higher among the elderly living alone, while the risk was not statistically significant among the elderly living with spouse. In contrast, Abas et al. [59], while comparing the depression of parents without migration of adult children, found that having all or some children migrated had lower levels of depression. Having all children out-migrated reduced depression compared to none or some children out-migrated [53]. Three studies [22, 28, 38] found no association between migration of adult children and the mental health of the elderly.

Among the studies reporting a bivariate association, three [18, 45, 58] reported higher prevalence of mental health problems for left behind parents while the remaining study [33] showed no significant association.

### Factors related to mental health status among the left behind parents

#### Gender

Eight studies examined the relationship between gender and mental health among the left behind elderly. Females had poorer mental health than males in five studies [12, 26, 33, 41, 50] while Xie et al. [48] observed women to be at lower risk. Gender differences were not observed in two studies [45, 46].

#### Age

Seven studies examined the influence of age on the mental health status of the left behind elderly and reported varied results. Multiple regression analyses showed cognitive ability and psychological health were negatively associated with age [26, 33]. In addition, Liu and Guo [60] found age was positively related with loneliness in a bivariate analysis. He et al. [41] reported the prevalence of depressive symptoms in the 71–80 years age group (45.2%) to be higher than the 65–70 years (37.4%) and >80 years (6.0%) age groups. Conversely, higher rates of loneliness [45] and anxiety/depression [46] were reported among the younger elders. No significant change in anxiety with increasing age was reported in a study conducted by Wang et al. [50].

#### Marital status/Type of residence

Marital status using marital status groups including currently married, never married, divorced, separated and widowed, was a frequently mentioned factor influencing mental health. Being (currently) married was associated with better mental health among the left behind elderly [26, 58, 62]. Similarly, living with a spouse decreased the risk of anxiety [50], depression [41], loneliness [45] and psychological ill health [48]. Two studies [33, 46] found no difference in mental health with respect to marital status.

#### Education

Seven studies assessed the relationship between education level and mental health with inconsistent results. Four [12, 41, 46, 50] indicated that left behind parents with higher educational level were less likely to develop mental health problems. Cheng et al. [45] reported a lower mean loneliness score among elderly with secondary education. However, Liu and Guo [62] found a higher level of education had a higher level of loneliness for left behind with higher levels of education. Guo et al. [33] reported no difference in mental health symptoms across different education groups.

#### Economic status

Seven studies addressed the association between economic status (measured mostly in terms of monthly or yearly income and self-perceived income) and mental health of the left behind elderly with all observing higher income related with lower levels of mental health disorders. The results of bivariate analyses showed that elderly in the lower income groups reported higher scores of anxiety [50]. In addition, low income was associated with higher levels of loneliness [45, 62], lower life satisfaction [60], and poorer mental health symptoms [33]. Similarly, low levels of self-perceived income was identified as a significant predictor of depression [55, 58]. Furthermore, He et al. [41] found a lower prevalence of depression among the elderly who had higher levels of financial support. Two studies used occupation as an economic indicator. Cheng et al. [45] reported a higher loneliness mean score among farmers compared to other occupations; however, the association was not significant under multiple regression. Similarly, skilled workers had the lowest mean anxiety score with the highest among farmers [50].

#### Place of residence

Three studies assessed the association between place of residence (urban or rural) and mental health with two reporting improvements for those in urban areas. Wang et al. [50] found higher anxiety scores among rural left behind elderly parents, and Su et al. [55] reported a lower prevalence of depression in urban residents. However, one study [33] showed no significant difference in mental health symptoms by place of residence.

#### Disease condition

Chronic disease(s) was associated with poor mental health conditions [33], depression [41] and lower levels of life satisfaction [60]. Su et al. [55] identified physical illness as a significant risk factor for depression, while Cheng et al. [45] found no association between chronic disease and loneliness.

#### Social support

Four studies measured social support using the Social Support Rate Scale (SSRS) [67] comprising three dimensions: objective support, subjective support and support utilization. Cheng et al. [45] reported significantly lower social support for left behind parents and found ‘objective support’ was a strong negative predictor of loneliness. Xie et al. [58] found all three dimensions of SSRS were negatively correlated with depression, but in the multivariate regression, only the dimension of ‘support utilization’ was significant. Social support was negatively associated with life satisfaction [60] and positively associated with loneliness [62]. Cheng et al. [45] also found that the social support from family as measured by Perceived Social Support from Family Scale (PSS-Fa) [68] and social interaction (as measured by WHOQOL-BREF) were negatively associated with loneliness.

#### Other reported factors

Higher levels of exercise and physical activity were found to improve cognitive function and psychological health [26], and reduce depression [41, 55] among left behind elderly parents. Increased frequency of the children’s visits was positively associated with mental health. Left behind parents whose children visited more often had lower depression [41] and better psychological health [48]. Xie et al. [58] identified religious belief as a risk factor for depression. Better relationships with children was also associated with higher levels of life satisfaction [60].

## Discussion

### The association between left behind status and mental health

The primary objective of this review was to identify the association between migration of adult children and the mental health of elderly parents left behind. The study designs were mostly cross sectional. While this study design limits causal inference, the quality assessment based on the JBI checklist for cross sectional analytical studies found most to be of high methodological quality allowing for adequate assessment of associations. The results were relatively consistent, where being left behind was negatively associated with mental health in 10 of the 16 studies with only 2 finding a positive association. The qualitative study [64] also found parents with adult children migrated experienced higher level of loneliness and depression.

Those left behind experienced higher levels of depression, loneliness, cognitive impairment, anxiety and had lower scores on psychological health compared to older parents with no migrant children. In a meta-analysis of studies concerning quality of life of the empty nest elderly by Lv et al. [69] found that mental health among the empty nest elderly was poorer than non-empty nesters.

In developed countries with higher standards of living and systems for social protection in older adults, independent living is often preferred [70]. In developing countries without social security and other welfare supports for older adults, intergenerational extended family is crucial for elderly health and well-being [71]. In South East Asian cultures, residing with adult children demonstrates ‘filial piety’ [72, 73]. The majority of studies included in this review were conducted in countries where filial piety is the major guiding principle and a strong intergenerational relationship is important. Older adults had emotional ties and high expectation for their children to provide physical, financial, instrumental and emotional support. Often when they are older, parents want to live with their children so that they can receive daily assistance and support. This may contribute to positive mental health and well-being. Being left behind may make them feel abandoned, and experience emotional ambivalence, anger and distress [74]. Older parents living with their children are reported to receive better daily care and support leading to better health [15].

A number of studies reported positive associations between parent-child co-residence and the mental health of older parents. Older adults who were left behind by migrant children were more susceptible to psychological distress such as depression [75]. Intergenerational co-residence has shown to be protective in many countries in different populations including Korea [76], Japan [77], China [78, 79] and Vietnam [80]. In Spain, Zunzunegui et al. [81] showed elders living with their children had more instrumental and emotional help and improved physical and mental health. Left behind adults in Sri Lanka had a higher prevalence of depression, anxiety and somatoform disorder [82]. Those left behind elderly may also feel a loss of status and fear for their future [83]. Cheng and Chan [84] demonstrated an association between filial behaviour of children and psychological well-being among Chinese older parents. Living with their son is considered the traditional living arrangement, but those living with their daughters report better psychological health [15, 77]. Unfortunately, no studies in our review reported the sex of the migrant children.

A study in India showed that living in multigenerational households had protective benefits in physical health [85]. Other studies showed older adults with migrant sons were more likely to report lifestyle-related chronic diseases such as hypertension, diabetes and heart disease [86, 87]. For those left behind, research shows increased time spent on agricultural and domestic work [88], especially among older women. Zhou et al. [89] observed lower utilization of healthcare services among the empty nest elderly. Liu et al. [18] emphasized that despite having ill health, empty nesters were more likely to report being unable to obtain the health care needed. Inadequate access to health care is likely to adversely affect mental health, given the relationship between physical and mental health disorders [90]. However, co-residence is not always influenced by parents needs. A study in China [91] emphasized parental support strongly influencing children to live with their parents.

Two of the 16 studies in this review (both from Thailand) [53, 59] showed improved mental health for those left behind, whilst four reported null findings. Children, who are leaving, are more likely to feel that their parents have an alternative means of support with most families having more than one child who can provide emotional, physical and financial support. A study by Stohr [92] showed that children in Moldova made strategic migration decisions to ensure some children stayed behind to care for their parents. Other children increase their contribution to compensate for their migrant siblings [93], and hence the effects of high rates of out-migration may be mitigated by this support [94].

Older parents with only some of their children migrated may not experience all the negative consequences compared to those with all their children migrated. These circumstances allow financial support from the migrant child and local support from the child(ren) at home which may have positive outcomes for their mental health and well-being. In addition, technological developments, especially in communication, have enabled continuous communication between the left behind parents and migrant children, potentially decreasing the negative impact of adult child migration [95, 96]. According to White and Edwards [21] empty nest status improved marital happiness; termed the ‘post-launch honeymoon’. The departure of the last child from the household can have a positive impact for parents [20]. The impact of left behind on the mental health of the elderly also depends on the socio-cultural context of the families. Mitchell and Lovegreen [97] reported higher levels of empty nest syndrome among the Indo/East Indian parents compared to British parents. Indian parents found more difficulties due to their expectations that sons stay with the parents and daughters remain until marriage. Gao et al. [26] found that that “living resources” and “availability of medical treatment” have an important mediating role in urban areas while engagement in “social activities” showed significant mediators among the rural sample.

### Risk factors of mental health disorders among the left behind elderly

This review also examined risk factors of mental health disorders among the left behind elderly. Fourteen factors were identified with different levels of influence, of which nine factors were associated with mental health disorders across the studies. The risk factors identified among the left behind elderly in this study are common to the elderly more generally. As there is a higher prevalence of mental health disorders for this cohort, consideration should be given to those most at risk.

Currently married older people had better mental health consistent with other studies showing widowhood negatively associated with subjective well-being [98] and mental health [99]. Living with a spouse was beneficial in reducing loneliness [100, 101] and Turner and Brown [102] noted co-residence with a spouse to be an important source of social support decreasing the risk of depression. For Buber and Engelhardt [14] the presence of a spouse or partner was more important than living with, or having regular contact with, their children. Paúl and Ribeiro [103] supported this observation as non-married status and/or widowhood lacked the support provided by a partner and sharing of intimate feelings that may result in loneliness. Empty nest couples have to rely on each other with spouses often providing essential daily care and emotional support [104].

Females may be at higher risk for mental health disorders consistent with other studies reporting older women at greater risk of loneliness [100] and depression [105]. Mothers often have a different bond with their child due to the time and effort they invest in raising their children. In contrast, males are more often engaged in social activities [13] reducing their loneliness whereas women whose main role is domestic, may be limited from establishing and maintaining non-family contacts [106].

The left behind elderly with lower education may be at greater risk of mental illness. This review supports the finding that educated empty nesters had greater subjective well-being [98] and cognitive function [107]. Lower education is associated with greater risk of depression [108, 109], dementia [110] and loneliness [100]. In general, educated older people are more likely to access health services [111] and seek new social contacts, thereby improving mental health.

Higher income was associated with better mental health consistent with research reporting higher levels of income associated with lower depressive symptoms [108, 112], improved quality of life [113] and decreased loneliness [100]. Lund et al. [114] reported a strong correlation between poverty and common mental health disorders. Higher income elderly are more financially independent and hence can pay expenses, and afford social activities, which may contribute to improved mental health and well-being [115]. Financial constraints may negatively affect self-esteem and self-efficacy, reducing social contacts.

Four out of five studies identified physical health as a risk factor for mental health problems with the other study reporting no association. Huang et al. [116] similarly found that chronic conditions such as stroke, cardiac/lung disease and loss of hearing/vision were risk factors for depression among older people. Other evidence [108, 109] shows that chronic disease is associated with poor psychological health consistent with our review results.

Physical exercise is noted to be beneficial for the elderly, with several studies finding significant psychological and cognitive benefits from regular physical activity [117–119]. A systematic review and meta-analysis of randomized control trials showed that exercise was associated with significantly lower depression in older people [120]. Exercise training was found to increase fitness, physical function, cognitive function, and positive behaviour in people with cognitive impairments [121].

Family and social support is a predictor of better mental health among the left behind elderly. Studies demonstrate the preventive effect of family and social support on depression [122], cognitive impairment [107] and loneliness [123]. Ryan and Willits [124] observed that the quality of relationships with spouse, children, and other family members was associated with feelings of well-being, rather than the quantity of relationships with the presence of family members not necessarily ensuring social support. The absence of positive relations with children is related to depression [125] as social support provides a buffering role [99]. Social support has direct as well as mediating effects among the elderly with mental health status and personality influencing the availability and perception of social support [102]. Intergenerational social support networks are important predictors of old-age health and survival in developing countries [126, 127]. Older adults who participate in socially engaging activities and have social support networks are less likely to become cognitively impaired than non-engaged older adults [128].

Four out of seven studies identified older age as a predisposing risk factor for mental health problems. Previous studies have shown that social activities decrease with age, which is a risk factor for depression [129]. Higher levels of loneliness [100] and depression [116] were reported with increased age among older adults as they reduced opportunities for social contact due to physical limitations and loss of close friends and family members [106].

Three studies compared the mental health of rural and urban elderly left behind with two finding those living in urban areas at lower risk while the remaining study found no difference. Rural people often have closer neighbourhood relationships than urban people, which may help to improve psychological well-being [130]. However, our findings favour urban inhabitants. This could be due in part to farming being important in the daily life of rural elders, with the out-migration of adult children directly affecting older parents’ workloads.

Of the 16 studies that examined the associations between migration of adult children and psychological well-being of the left behind elderly, only four employed longitudinal design. Three of the four longitudinal studies reported increased risk of psychological ill health among the parents with migrant children. The cross sectional design of the majority of studies limits the ability to determine cause and effect relationships [131] hence the association between the adult child’s migration and the mental health outcomes of older parents, conceivably due to reverse causality. The decision to migrate may be influenced by the health status of elderly parents. Children may be more likely to migrate if the older parents are in good health and they have strong family and social support networks. Conversely, adult children with elderly parents with poor health may migrate to pursue higher earnings to help pay for medical expenses. Migrants and their families may have better education, higher access to socioeconomic resources or social capital [132], and these characteristics may contribute to better health outcomes of the elderly parents irrespective of the children’s migration [16, 133].

### Policy recommendations

The findings of this review have important implications for programs and policies aiming to promote the mental health of older adults. Targeting social security for the elderly left behind could enhance the feeling of security and support, thereby improving metal health and well-being [123]. Given the higher prevalence of physical illnesses and chronic diseases among the left behind elderly and its association with mental disorders, it is recommended to consider this risk group in health service delivery. The health care delivery system in low-income countries is inadequate to meet the mental health needs of older people [134–136] resulting in a range of unmet emotional and physical needs among the older adults left behind.

Programs to extend emotional intimacy between older parents and their migrant children are required, with intergenerational relationships and translational care particularly important in reducing risk of mental illnesses among the older adults. Zechner [137] enlisted the three basic elements of transnational care: distance, resources and circumstances. Attention should be paid to the social policies involved in care-related activities. Maintaining older parents’ contact with their migrant children, being visited by children more frequently, and engaging older people in a range of social activities reduces the negative consequences of their children’s migration [138]. Migrant children can provide emotional support or may organize the care needs of the older parent(s) with someone who lives close by [137]. Certainly, the availability of social media and communication technologies provides opportunities for more active communication and interaction within the family irrespective of geographical location. Consideration should be given to training community health workers and field workers in identifying older adults who are at risk, connecting to community resources to those who are at risk and counselling families to better support close family relationships.

Efforts to lower the prevalence of mental health disorders in the left behind elderly should target those at particular risk. Special attention should be given to the elderly who are unmarried or widowed, have lower education, poorer socioeconomic background, older, living in rural areas and with chronic disease.

Finally, physical activity plays an important role to offset the negative influence of an empty nest on health and well-being. A greater focus on the importance of physical activity levels by both professionals and volunteers [139] may promote and support physical activities for the left behind elderly.

### Implications for future research

A number of implications for future studies for the mental health of left behind elderly arise from this review. Family support plays a pivotal role in determining the psychological well-being of the older parents. While the migration of the younger generation is unavoidable in many societies, its effect is often to undermine traditional care and support structures for older parents. Hence more research is required to address care and support needs from friends, neighbours and other community based organizations. Such studies should also examine the effects of different types of social support to improve the mental health status among older adults left behind.

The issue of transnational care; care giving across political and geographical spaces, is not well recognized in gerontology [137, 140, 141]. Future studies are required to identify effective transnational care provision. Well-designed studies are also required to identify additional factors related to mental health among the left behind elderly, as this review did not identify the effect of important risk factors such as remittances, frequency and intensity of the communication between parents and migrant children, purpose of migration, migrant receiving place or country, physical environment (e.g. housing) in which elderly were residing, religious belief, functional disability and bereavement or loss of close contacts by the elderly. In particular, information technology and religious attendance are likely to have a positive effect on mental health and increased social relationships among the elderly [142]. Future research could also compare systematic differences in the risk factors of mental health disorders between the left behind and non-left behind older adults.

Longitudinal studies are required to provide clarity on the direction of causality between migration of adult children and mental health of elderly parents left behind. Apart from the longitudinal studies, a matched-control design with parents whose children emigrated with those with children living nearby would help to distinguish the empty nest component from the left behind. Qualitative studies are essential to understand diverse and complex sociocultural contexts. Local surveys and investigations will also inform local service needs.

### Study limitations

This review is not without limitations. The definition of ‘left behind elderly’ varied across the studies and many different definitions of mental health are summarised in this review. Studies were diverse and often did not report prevalence of any aspects of mental health, nor the strength of association for each risk factor. The high level of heterogeneity among the studies precluded meta-analysis.

Results of the multivariate analyses might be convoluted by adjustments for different variables in different studies. Likewise, only the main effects of risk factors on mental health disorders were reviewed and as such, it is not clear whether the concurrent occurrence of multiple risk factors results in a synergistic increase in the risk.

The studies included in this review did not always measure potential risk factors that could have affected the mental health of the left behind elderly and often only provided bivariate analyses, making it difficult to confirm the association between migration of adult children and the mental health of parents left behind under the influence of potential confounders. In addition, risk factors for mental health disorders identified in this review are based on studies reporting risk factors from left behind elderly. Comparison of putative risk factors between left behind and non-left behind groups would be more informative.

The review did not assess publication bias, with negative or non-significant results being less likely to be submitted and accepted for publication [143]. Other limitations of this review include the search was limited to peer-review articles published in English with grey literature excluded. Many studies employed secondary analyses of large samples, which may have produced statistically significant results for effect sizes which are small, limiting the clinical significance of the results. Almost half of the studies included in this review are from China. This may reflect a general lack of research in other low-income countries, which is unfortunate given the potentially higher vulnerability of older people being left behind and psychological disorders [144].

## Conclusion

The key finding of this review is that being left behind is negatively associated with the mental health of older adults. Empty nesters were at higher risk of mental health disorders such as loss of cognitive function, depression, anxiety and loneliness. Elderly living with their children may receive better care, economic and emotional supports. The risk factors for mental disorders include marital status, income, education, physical health status, gender, age, family and social support, and physical exercise.

This study synthesises the research related to mental health of the left behind elderly parents, thereby advancing our theoretical and empirical understanding of out-migration of adult children and its implication on psychological well-being of the parents. Authorities and organizations working in the field of gerontology should be aware that the left behind elderly are at increased risk of mental health problems. More responsive preventive measures and effective management approaches are required for this cohort. More rigorous studies are required to identify the additional risk factors of mental health problems using clinically relevant instruments. Additionally, mechanisms of transnational care by the migrant children should be explored to reduce the psychological cost of the phenomena of being ‘left behind’.

## Supporting information

S1 Table Quality assessment of included studies. (DOCX)

S1 Checklist PRISMA checklist. (DOC)
