# Supplementary material for: Migration of adult children and mental health of older parents ‘left behind’: An integrative review
Source: PLoS One. 2018 Oct 22;13(10):e0205665. doi: 10.1371/journal.pone.0205665 (PMC6197646; doi:10.1371/journal.pone.0205665)
Supplement: S1 Table — (DOCX) [file pone.0205665.s001.docx]

**S1 Table: Quality assessment of included studies**

| **SN** | **Studies** | **1. Were criteria for inclusion in the sample clearly defined?** | **2. Were the study subjects and the setting described in detail?** | **3. Was the exposure measured in a valid and reliable way?** | **4. Were objective, standard criteria used for measurement of the condition?** | **5. Were confounding factors identified?** | **6. Were strategies to deal with confounding factors stated?** | **7. Were the outcomes measured in a valid and reliable way?** | **8. Was appropriate statistical analysis used?** |
| --- | --- | --- | --- | --- | --- | --- | --- | --- | --- |
| 1 | Gao et al. [26] | Yes | Yes | Yes | Yes | Yes | Yes | Yes | Yes |
| 2 | Waidler et al. [28] | Yes | Yes | Yes | Yes | Yes | Yes | Yes | Yes |
| 3 | Mosca and Barrett [30] | Yes | Yes | Yes | Yes | Yes | Yes | Yes | Yes |
| 4 | Guo et al. [33] | Yes | Yes | Yes | Yes | Yes | Yes | Yes | Yes |
| 5 | Downer et al. [35] | Yes | Yes | Yes | Yes | Yes | Yes | Yes | Yes |
|  | Antman [12] | No | Yes | Yes | No | Yes | Yes | No | Yes |
| 6 | Chang et al. [38] | Yes | Yes | Yes | Yes | Yes | Yes | Yes | Yes |
| 7 | He et al. [41] | Yes | Yes | NA | Yes | Yes | Yes | Yes | yes |
| 8 | Böhme et al. [22] | Yes | Yes | Unclear | Yes | Unclear | Unclear | Unclear | Unclear |
| 9 | Zhai et al. [43] | Yes | Yes | Yes | Yes | Yes | Yes | Yes | Yes |
| 10 | Cheng et al. [45] | Yes | Yes | Yes | Yes | Yes | Yes | Yes | Yes |
| 11 | Liang and Wu [46] | Unclear | Yes | Yes | Yes | Yes | Yes | Yes | Yes |
| 12 | Xie et al. [48] | Yes | Yes | NA | Yes | Unclear | Unclear | Yes | Unclear |
| 13 | Sekhon and Minhas [49] | Yes | Yes | No | No | No | No | No | No |
| 14 | Wang et al. [50] | Yes | Yes | NA | Yes | Yes | Yes | Yes | Yes |
| 15 | Abas et al. [53] | Yes | Yes | Yes | Yes | Yes | Yes | Yes | Yes |
| 16 | Su et al. [55] | Yes | Yes | NA | Yes | Yes | Yes | Yes | Yes |
| 17 | Adhikari et al. [56] | Yes | Yes | Yes | No | Yes | Yes | No | Yes |
| 18 | Sun et al. [57] | Yes | Yes | Yes | Yes | Yes | Yes | Yes | Yes |
| 19 | Xie et al. [58] | Yes | Yes | Yes | Yes | Yes | Yes | Yes | Yes |
| 20 | Abas et al. [59] | Yes | Yes | Yes | Yes | Yes | Yes | Yes | Yes |
| 21 | Liu and Guo [60, 62] | Yes | Yes | Yes | Yes | Yes | Yes | Yes | Yes |
| 22 | Liu et al. [18] | Yes | Yes | Yes | Yes | No | No | Yes | Yes |
| 23 | Miltiades [64] | NA | NA | NA | NA | NA | NA | NA | NA |
